# Advancing methods in big data capture, integration, classification and liberation

**DOI:** 10.1186/s13104-023-06340-z

**Published:** 2023-04-27

**Authors:** Eftim Zdravevski, Ivan Miguel Pires

**Affiliations:** 1 Faculty of Computer Science and Engineering, University Ss Cyril and Methodius, Skopje, 1000 Macedonia; 2 Instituto de Telecomunicações, Universidade da Beira Interior, Covilhã, 6200-001 Portugal; 3 Polytechnic Institute of Santarém, Santarém, Portugal

## Abstract

This special issue focuses on the importance of advancing research techniques for managing and analyzing data in today’s data-rich landscape. In this editorial, we set the context and invite contributions for a *BMC* Collection of articles titled ‘Advancing methods in data capture, integration, classification and liberation’. The collection emphasizes the need for efficient ways to standardize, cleanse, integrate, enrich, and liberate data, highlighting recent advancements in research methods and industrial technologies that facilitate this. We invite researchers to submit their best work to the collection and to showcase the latest advancements and additions to research techniques.

## Main text

The exponential growth of data presents both opportunities and challenges [1]. Data can provide valuable insights and lead to new discoveries. Still, the sheer volume of data generated daily can be overwhelming and challenging to manage, process, and use [2].

Over the span of its use, any data set must undergo a series of crucial processes for it to be utilized successfully. Firstly, data must be captured from a given source and stored in a usable format. In turn, data sets from varying sources may need to be integrated and consolidated, increasing the value, accuracy, or reliability of the answers we seek to gain [2]. Next, data classification can help improve data usability and discoverability. This process refers to categorizing or labeling based on some pre-defined criteria or based on some automatically identified patterns or relationships by machine learning processes.

Furthermore, the process of making data more accessible, usable, and shareable is often encompassed by data liberation. This term can implicate removing data access or licensing restrictions, converting data into open or standardized formats, or making data available through APIs or other interfaces that facilitate integration and analysis. Data liberation aims to enable more people to use data to gain insights and make informed decisions. These challenges are relevant for open and reproducible research. Likewise, this has significant implications for organizations with siloed data and business users who require instant access to data and insights from it [3].

The recent growth of sensor technologies has made it possible to capture real-time data in various new contexts, including wearable devices, manufacturing, smart homes, smart cities, autonomous vehicles, smart agriculture [4] and medicine [5], to name a few. Moreover, these advancements coincide with the exponential growth in machine learning applications [6], causing completely novel use cases to emerge and raising new challenges. This collection seeks to publish research note articles that address novel or improved data capture methods, better processes for data classification and contextualization, methods for integrating data from a variety of sources, improving automatic tagging of data, identifying sensitive information, recognizing relationships between data, and more efficient ways for users to view and export their data. With the vast choices of technology stacks, significant challenges exist in minimizing the total cost of ownership of cloud infrastructures and choosing optimal, cost-effective solutions [7].

We are also seeking articles that report improvements to existing methods and techniques or introduce new methodologies to the field. For example, novel data capture techniques could include the use of machine learning algorithms for automated data acquisition or the use of wearable devices to collect data in real-time. Improvements in data classification and contextualization could involve more sophisticated natural language processing techniques, while the integration of data from multiple sources may require new approaches to data modeling and data warehousing. Finally, better methods for data liberation could include more user-friendly interfaces or improved tools for data visualization.

We also welcome commentaries discussing the history and future of significant techniques or methodologies in this field as well as the controversies they may present and how they can be addressed. These commentaries should provide a thoughtful and well-informed perspective on the relevant issues.

We invite researchers to submit their work to this collection, which promises to showcase the latest advancements and additions to research techniques in the data science field. With the rapidly changing landscape of data science, we believe that this collection will contribute to a deeper understanding of the field and facilitate further research in this critical area. We hope that the papers published in this issue will lead to a better understanding of the challenges and opportunities that researchers face when working with data and inspire new and innovative techniques for data management and analysis.

## Data Availability

Considering that this article is a Guest Editor Editorial, and there is no data and material used in its preparation, there is nothing to declare in this section.
